# New and Emerging Drug Therapy in Primary Open-Angle and Normal-Tension Glaucoma: Pharmacology, Neuroprotection and the Endpoint Problem

**DOI:** 10.3390/ph19101598

**Published:** 2026-10-09

**Authors:** Stephanie Mroczkowska, Leanne Smewing, Doina Gherghel

**Affiliations:** 1 Eye and Vision Research Group, Faculty of Health, University of Plymouth, Plymouth PL4 6AB, UK; 2 Dompé Farmaceutici S.p.A, Via Santa Lucia, 6, 20122 Milan, Italy

**Keywords:** primary open-angle glaucoma, normal-tension glaucoma, neuroprotection, intraocular pressure, retinal ganglion cell, visual field, clinical trial endpoints, sustained drug delivery, nicotinamide

## Abstract

Glaucoma is a group of progressive optic neuropathies characterized by retinal ganglion cell (RGC) loss, optic nerve head damage and corresponding visual-field loss. Intraocular pressure (IOP) is the principal modifiable risk factor, and lowering IOP remains the only treatment approach that has been shown in randomized clinical trials to slow glaucomatous progression, including in normal-tension glaucoma (NTG). However, IOP reduction does not eliminate progression in all patients, indicating that susceptibility to pressure-related damage is heterogeneous and that additional biological mechanisms are relevant. This review examines current and emerging pharmacological strategies for IOP lowering, approaches intended to protect RGCs independently of IOP, and the methodological problem of demonstrating neuroprotection in clinical trials.

## 1. Introduction

Glaucoma is a heterogeneous group of chronic optic neuropathies in which progressive RGC dysfunction and loss lead to structural damage of the optic nerve head and corresponding visual-field impairment. Although IOP is not the only determinant of disease, it remains the principal modifiable risk factor and the only therapeutic target for which lowering has consistently demonstrated a reduction in the risk of glaucomatous progression [1,2,3].

This is particularly important in NTG. The Collaborative Normal-Tension Glaucoma Study showed that a 30% reduction in IOP reduced the proportion of treated eyes reaching predefined progression endpoints compared with untreated controls. Thus, normal-range IOP should not be interpreted as absence of pressure-related risk; rather, it demonstrates that the relationship between IOP and damage varies substantially between individuals [4]. Recent longitudinal evidence reinforces this concept. In the UK Glaucoma Treatment Study, patients showed substantial variation in the rate of structural and functional deterioration across the range of IOP, indicating that IOP alone does not fully explain an individual’s susceptibility to glaucomatous damage [5]. This variability is consistent with evidence that the effect of IOP is influenced by the biomechanical properties of the optic nerve head, including the lamina cribrosa, and ocular perfusion. These factors may influence how an individual eye responds to a given level of IOP. Thus, the same IOP may confer substantially different risks of glaucomatous damage between eyes [5,6,7,8].

These observations have led drug development in two related directions. One seeks to improve the magnitude, duration and consistency of IOP lowering, including approaches that reduce dependence on daily adherence. The other seeks to increase the resilience of RGCs and their axons to injury. These approaches address different aspects of the disease process: IOP-lowering therapies act on a modifiable risk factor with established effects on glaucomatous progression, whereas pressure-independent approaches seek to modify the tissue response to injury. Demonstrating that the latter strategy can preserve visual function in patients, however, has remained substantially more difficult [1,2,3].

## 2. Lowering the Intraocular Pressure

Topical glaucoma medicines lower IOP primarily by reducing aqueous humour production or increasing aqueous outflow through the trabecular and/or uveoscleral pathways. Treatment is individualized around a target IOP that reflects baseline pressure, the extent of existing damage, rate of progression, life expectancy and other risk factors. Because there is no single IOP level that is universally safe for every glaucomatous eye, effective treatment depends not only on achieving sufficient pressure reduction but also on maintaining that reduction over time [1].

Current drug development has therefore focused on two practical aspects of IOP lowering: improving the pharmacological effect achieved by treatment and improving the way treatment is delivered. The first has led to agents that target additional or complementary mechanisms of aqueous outflow. The second has led to sustained-release approaches designed to maintain drug exposure while reducing the frequency of administration.

### 2.1. Newer Pharmacological Approaches

Prostaglandin FP-receptor agonists remain a major first-line pharmacological approach because of their established efficacy, once-daily dosing and extensive clinical experience. More recent agents have sought to complement prostaglandin-mediated outflow enhancement by targeting additional pathways, including the trabecular meshwork, while also addressing the duration and consistency of IOP lowering. Among these, sepetaprost is a prodrug whose active metabolite acts as a dual FP/EP3 receptor agonist. In the ANGEL phase IIb study, the 0.002% dose produced IOP lowering comparable to latanoprost, although that study was not designed to establish non-inferiority. Subsequent phase III data from ANGEL-J1 demonstrated non-inferiority of sepetaprost 0.002% to latanoprost 0.005% for mean diurnal IOP at week 4, with non-inferiority also maintained over 3 months. Sepetaprost was approved in Japan in 2025 as Setaneo. Conjunctival hyperaemia was the most common adverse drug reaction in ANGEL-J1, occurring more frequently with sepetaprost than latanoprost. The available evidence therefore supports sepetaprost as an alternative prostaglandin-based IOP-lowering therapy but does not establish a neuroprotective effect or superior long-term visual-field preservation [9,10,11].

Nitric oxide (NO)-donating prostaglandin conjugates have been developed to target both prostaglandin-mediated outflow and the conventional trabecular pathway. Two such agents are latanoprostene bunod and NCX 470.

Latanoprostene bunod combines an FP-receptor agonist with an NO-donating moiety. The NO component is intended to enhance conventional trabecular outflow through the NO–soluble guanylate cyclase–cGMP pathway, complementing the uveoscleral effect of FP activation. In the VOYAGER trial it produced a modestly greater diurnal IOP reduction than latanoprost [12].

NCX 470 combines bimatoprost with an NO-donating group. In the 691-patient MONT BLANC phase III trial, NCX 470 0.1% lowered IOP more than latanoprost at all six evaluated time points; the difference was statistically significant at four of six time points. The trial met its non-inferiority primary endpoint. A second phase III trial, DENALI, also met its primary objective, with statistical superiority at three of six time points and numerical superiority at five of six. As of July 2026, an NDA for NCX 470 had been submitted in the United States. These data support continued development of the drug as an IOP-lowering therapy; whether its nitric oxide–donating component provides any additional clinical benefit beyond IOP lowering is a separate question that was not addressed by these trials [13,14,15].

Traditional prostaglandins and aqueous suppressants leave the diseased trabecular meshwork (TM) largely unaddressed. By modulating Rho kinase signalling and the cytoskeleton of trabecular meshwork and Schlemm’s canal cells, ROCK inhibitors reduce resistance to aqueous humour outflow [16]. Netarsudil is a ROCK inhibitor approved for the reduction of elevated IOP in patients with open-angle glaucoma or ocular hypertension. Its FDA prescribing information identifies increased trabecular outflow as the principal mechanism by which netarsudil is believed to lower IOP, while experimental and clinical literature also describes effects on episcleral venous pressure and aqueous humour dynamics. Conjunctival hyperaemia remains a common adverse effect.

Omidenepag isopropyl is a selective EP2-receptor agonist and a non-prostaglandin option, suitable for patients exhibiting intolerance to traditional FP agonists. In the AYAME phase III study it was non-inferior to latanoprost for IOP lowering. The clinical literature and product information recommend caution regarding macular oedema in susceptible pseudophakic/aphakic eyes [17].

The key agents and their comparative evidence are summarized in Table 1.

The pharmacological differences are also relevant to exposure and tolerability. Sepetaprost is a prodrug whose active metabolite, ONO-AG-367, acts at FP and EP3 receptors; PMDA clinical pharmacology data support once-daily topical dosing. Latanoprostene bunod is rapidly converted in the eye to latanoprost acid and butanediol mononitrate; after repeated topical administration, latanoprostene bunod itself was not quantifiable in plasma, while latanoprost acid appeared rapidly, with a mean Tmax of approximately 5 min. Netarsudil is converted by ocular esterases to its active metabolite AR-13503, with no quantifiable plasma netarsudil in a multiple-dose study and only minimal detectable systemic exposure to the metabolite. Omidenepag isopropyl is similarly a prodrug, rapidly hydrolysed in the eye to omidenepag; plasma Cmax occurred at approximately 10–15 min and there was no accumulation after 7 days of once-daily dosing. These pharmacokinetic profiles favour predominantly local ocular exposure, but local tolerability remains an important limitation. Conjunctival hyperaemia is common with sepetaprost, latanoprostene bunod, netarsudil and omidenepag, while netarsudil is additionally associated with corneal verticillata and, in post-marketing experience, epithelial corneal oedema in susceptible eyes. Omidenepag is associated with ocular inflammation and macular oedema as recognised warnings. Thus, the advantage of newer agents is not simply additional pharmacology; it is the possibility of achieving adequate IOP lowering through complementary pathways while retaining a dosing frequency and safety profile that are acceptable for long-term treatment [11,18,19,20].

**Table 1:** Selected newer IOP-lowering approaches.

Agent	Development/Evidence	Comparator	Key Evidence	Status (2026)
Sepetaprost (FP/EP3 agonist)	ANGEL phase IIb; ANGEL-J1 phase III	Latanoprost	Phase III ANGEL-J1 demonstrated non-inferiority of 0.002% sepetaprost to 0.005% latanoprost for mean diurnal IOP; conjunctival hyperaemia was the most common ADR [9]	Approved in Japan (2025)
Latanoprostene bunod (FP agonist + NO donor)	VOYAGER	Latanoprost	Modestly greater diurnal IOP reduction [12]	Approved in the US
NCX 470 (NO-donating bimatoprost)	MONT BLANC phase III; DENALI phase III	Latanoprost	Non-inferior in both pivotal trials; numerical superiority at most/all time points with statistical superiority at selected time points [13,14,15]	U.S. NDA submitted July 2026
Netarsudil (ROCK inhibitor)	ROCKET phase III	Timolol	Effective IOP lowering; conjunctival hyperaemia common [21]	Approved
Omidenepag isopropyl (EP2 agonist)	AYAME phase III	Latanoprost	Non-inferior IOP lowering [17]	Approved in several markets

### 2.2. Sustained-Release and Reduced-Frequency Delivery

Topical treatment can be effective yet difficult to sustain when control depends on repeated daily administration and is limited by patient adherence. Sustained-release delivery approaches aim to maintain therapeutic drug exposure over longer periods while reducing the frequency of administration and reliance on patient-administered eye drops. The objective is to improve the consistency of drug exposure while reducing the burden of repeated topical treatment [22,23,24].

Several delivery platforms have been investigated for glaucoma, including intracameral implants, punctal delivery systems, conjunctival inserts and rings, drug-eluting contact lenses, subconjunctival depots, microspheres, nanospheres and other depot systems (Table 2). These approaches differ in drug-loading capacity, duration of release, route of administration, invasiveness, retention and reversibility, and each has distinct safety and feasibility considerations [22,23,25].

Intracameral implants are currently the most clinically advanced sustained-release approach. Durysta (bimatoprost intracameral implant) was approved by the US FDA for the reduction of IOP in patients with open-angle glaucoma or ocular hypertension. The biodegradable implant provides prolonged bimatoprost exposure following intracameral administration. However, repeated administration was associated with increased corneal endothelial cell loss in phase III studies, and the current US indication is limited to a single administration per eye [26,27].

iDose TR (travoprost intracameral implant) provides sustained travoprost delivery from a non-biodegradable implant anchored in the iridocorneal angle. In phase III studies, the implant provided sustained IOP lowering and met the prespecified non-inferiority criteria against twice-daily timolol during the primary efficacy period [27]. In January 2026, the US FDA approved a supplemental application allowing re-administration of iDose TR under a repeat-treatment protocol in patients meeting specified corneal endothelial safety criteria [28].

The principal sustained-release platform characteristics are summarized in Table 2, while selected clinical products are summarized in Table 3.

The clinical translation of these platforms has not been uniform. Failure to enter or continue clinical development cannot be attributed to a single formulation limitation without programme-specific evidence. Relevant barriers include insufficient duration or reproducibility of drug release, inadequate intraocular exposure, dose-loading constraints, inflammatory or corneal toxicity, endothelial-cell loss, device retention or migration, reversibility, manufacturing and sterility requirements, and the difficulty of demonstrating a clinically meaningful advantage over effective topical therapy. A platform may therefore be scientifically viable while a particular programme is discontinued because the therapeutic index, delivery profile, manufacturing pathway or clinical benefit is not sufficient. Conversely, sustained exposure alone does not establish clinical value: the delivered drug must reach the relevant ocular compartment at an effective concentration for an appropriate duration without introducing a safety burden that offsets the adherence advantage [22,23,24,25].

Other sustained-release approaches have been investigated, including subconjunctival depots, microspheres, nanoparticles, hydrogels and other injectable systems. Some individual programmes have been discontinued, but these delivery technologies remain active areas of preclinical and translational research, with recent studies continuing to evaluate their potential to provide prolonged local drug exposure and reduce dosing frequency [22,24,25].

Sustained-release technologies address a limitation of conventional topical therapy that is distinct from the pharmacological mechanism of IOP lowering. The aim is to maintain effective drug exposure over an extended period while reducing the frequency and burden of administration. Their clinical value depends on whether sustained delivery can provide durable IOP control with an acceptable safety profile and a meaningful reduction in treatment burden compared with topical therapy. Effective and sustained IOP control, however, does not necessarily eliminate the risk of glaucomatous progression. This leads to a different question: whether additional therapeutic strategies are needed to protect the optic nerve and preserve visual function beyond IOP lowering.

## 3. Why Treat Beyond the IOP?

IOP lowering remains the principal established treatment for reducing the risk of glaucomatous progression, but it does not eliminate progression in all patients. Differences in the rate of damage between eyes exposed to similar levels of IOP further indicate that susceptibility to glaucomatous injury varies between individuals. These observations have prompted interest in therapeutic approaches that target RGC and axonal injury, with the aim of preserving neuronal function independently of further IOP reduction.

Normal-tension glaucoma (NTG) provides a particularly clear clinical illustration of this distinction. Patients can develop characteristic glaucomatous structural and functional damage without consistently elevated untreated IOP, yet IOP lowering still reduces the risk of progression. NTG therefore does not represent a purely pressure-independent form of glaucoma; rather, it illustrates that pressure-related injury and differences in susceptibility to injury can coexist. This has led to increasing interest in factors that may influence the vulnerability of the optic nerve to damage at a given level of IOP.

Vascular dysregulation is one potential contributor to this variability in susceptibility. Studies in NTG have identified abnormalities in ocular perfusion, systemic blood-pressure regulation and vascular reactivity. In a prospective longitudinal study, cumulative nocturnal hypotension was associated with subsequent visual-field progression in NTG, with the duration and magnitude of nocturnal blood-pressure reduction predicting VF loss [32]. Studies comparing newly diagnosed POAG and NTG have also identified overlapping ocular and systemic vascular abnormalities, including increased nocturnal blood-pressure variability, increased peripheral arterial stiffness and reduced ocular perfusion pressure [33].

Evidence of systemic endothelial dysfunction provides a further potential link between vascular regulation and glaucomatous susceptibility. Peripheral flow-mediated vasodilation was significantly impaired in patients with NTG compared with healthy controls, and subsequent work found impaired endothelial function in both NTG and POAG, with greater impairment in NTG [34,35]. A longitudinal study of patients with open-angle glaucoma also found an association between peripheral endothelial function and subsequent visual-field progression, although the study was small and included both NTG and POAG [36].

More recent imaging studies provide evidence directly linking ocular microvascular changes with progression. In a prospective cohort of 110 eyes with NTG, faster loss of peripapillary capillary vessel density measured by OCTA was associated with faster visual-field deterioration over longitudinal follow-up [37]. Together, these findings support a role for vascular dysregulation and impaired ocular microvascular function in susceptibility to glaucomatous injury, particularly in NTG, while also showing that these abnormalities are not unique to NTG and should not be regarded as a single causal mechanism.

Vascular mechanisms are unlikely, however, to account for all of the processes underlying RGC and axonal injury. Other pathways implicated in glaucomatous neurodegeneration include excitotoxicity, oxidative and metabolic stress, impaired neurotrophic support and disruption of axonal transport [38,39,40]. These processes may interact with pressure-related factors and with various vascular stressors rather than operate independently. The therapeutic question is therefore not simply whether IOP can be lowered further, but whether targeting mechanisms that influence neuronal vulnerability can provide additional protection against glaucomatous damage and preserve visual function.

### 3.1. Neuroprotective Strategies

The human evidence and principal limitations of the pressure-independent strategies discussed below are summarized in Table 4.

The limitations of IOP lowering have led to clinical investigation of treatments intended to preserve retinal ganglion cells (RGCs) and their axons through mechanisms additional to IOP reduction. These approaches have targeted different aspects of neuronal injury, including glutamatergic excitotoxicity, neurotrophic support, mitochondrial and metabolic dysfunction, vascular regulation and cellular stress. However, the clinical development of neuroprotection has demonstrated the difficulty of translating a biologically plausible mechanism or experimental effect into a treatment that measurably slows glaucomatous progression or preserves visual function (Table 4). Reviews of the field have highlighted this translational gap, and the failure of large phase III studies of memantine remains an important example [41].

**Table 4:** Pressure-independent/neuroprotective strategies.

Approach	Proposed Mechanism	Human Evidence	Main Limitation	Status
Brimonidine	α2-adrenergic; proposed neuroprotective effects	LoGTS: less VF progression signal vs. timolol [42]	Differential dropout/allergy limits interpretation	Unproven
Memantine	NMDA antagonism/anti-excitotoxic	Two phase III trials [43]	No significant reduction in progression	Negative phase III
Nicotinamide	NAD+ precursor/metabolic resilience	Human RCTs; electrophysiological signals [44,45]	No definitive VF-preservation evidence; high-dose safety concerns	Investigational
Ginkgo biloba	Vascular/antioxidant hypothesis	NTG crossover RCT [46]	No improvement in VF or contrast sensitivity	Negative RCT
Citicoline	Membrane/neuronal metabolism	Small human studies [47]	Electrophysiological effects not equivalent to VF preservation	Preliminary
CoQ10 + vitamin E	Mitochondrial/antioxidant	Small human studies [48]	No definitive long-term functional outcome	Preliminary
CNTF/NT-501	Neurotrophic support via sustained delivery	Phase I safety; randomized phase II studies [49,50,51]	Definitive efficacy not established	Investigational
BL1107	Topical α2-adrenergic agonist; proposed neuroprotection/neuroenhancement	Phase II in POAG/OHT; visual-field MD primary endpoint	Did not meet primary endpoint; development discontinued in 2026 [52]	Discontinued
Vascular approaches	Ocular perfusion/vascular modulation	Small human studies in NTG	Physiological effects without established progression benefit [46,53]	Unproven

#### 3.1.1. Memantine

Memantine was developed around the excitotoxicity hypothesis, which proposes that excessive glutamatergic stimulation of NMDA receptors contributes to intracellular calcium overload and RGC injury. The approach showed neuroprotective effects in experimental glaucoma models and progressed to two large, randomized phase III trials. A total of 2298 patients with open-angle glaucoma were randomized to memantine 10 mg, memantine 20 mg or placebo and followed for 48 months. Neither dose produced a consistent or clinically meaningful reduction in glaucomatous progression compared with placebo across visual-field and optic-nerve endpoints. The studies therefore did not establish a neuroprotective effect of memantine in glaucoma [43].

The memantine experience also illustrates a limitation of neuroprotection trials: the absence of a treatment effect may reflect failure of the intervention, limitations in target selection, insufficient sensitivity of the outcome measures, or a combination of these factors. This issue has become increasingly important as newer approaches are developed with more sensitive structural, functional and biological endpoints [41].

#### 3.1.2. Brimonidine

Brimonidine has been investigated for potential neuroprotective effects beyond its IOP-lowering activity, including effects on neurotrophic signaling and retinal neuronal survival. The Low-Pressure Glaucoma Treatment Study compared brimonidine 0.2% with timolol 0.5% in patients with low-pressure glaucoma and achieved similar treated IOP between groups. Fewer patients assigned to brimonidine developed visual-field progression among those who remained in the study. Treatment discontinuation because of ocular adverse events, however, was substantially more frequent with brimonidine, occurring in 28.3% of brimonidine-treated participants compared with 11.4% of timolol-treated participants. This differential discontinuation complicates interpretation of the apparent difference in visual-field progression. Brimonidine therefore provides a clinically relevant signal consistent with a possible IOP-independent effect, but the study did not establish brimonidine as a neuroprotective treatment for glaucoma [42].

#### 3.1.3. Vascular Approaches

Vascular mechanisms represent another approach to modifying susceptibility to glaucomatous injury, particularly in NTG. Human studies have demonstrated that pharmacological manipulation can alter ocular haemodynamics, but these physiological effects have not established disease modification. In a randomized, placebo-controlled crossover study of 14 patients with NTG, oral nimodipine increased optic nerve head and choroidal blood flow and produced a modest improvement in tritan colour contrast sensitivity two hours after administration. The changes in colour contrast sensitivity, however, were not correlated with the changes in ocular blood flow, and the study was not designed to assess long-term visual-field progression [53].

Ginkgo biloba has also been investigated because of proposed vascular and antioxidant effects. An early randomized crossover study in 27 patients with NTG reported improvement in visual-field indices after four weeks of treatment [54]. A subsequent randomized crossover trial in 35 patients with NTG, however, found no difference between Ginkgo biloba and placebo in visual-field mean deviation or contrast sensitivity [46]. The clinical evidence remains inconsistent and does not establish vascular manipulation or Ginkgo biloba as a treatment that slows glaucomatous progression.

#### 3.1.4. Nicotinamide and Metabolic Rescue

The bioenergetic hypothesis is currently one of the most active areas of clinical investigation. Experimental work has shown that declining NAD availability and mitochondrial dysfunction can increase RGC vulnerability, and that nicotinamide, a precursor of NAD, can protect RGC somata, axons and dendrites in experimental models [55,56].

Human clinical development has now progressed beyond short-term exploratory studies. A phase 2 randomized clinical trial evaluated oral nicotinamide and pyruvate in 42 patients with treated open-angle glaucoma. The intervention was associated with a greater number of visual-field locations showing improvement beyond expected test–retest variability and an improvement in PSD slope, but the study was short, with a median follow-up of approximately 2.2 months, and did not demonstrate a significant difference in the rate of change in MD or VFI [57].

A 2026 randomized crossover trial provides more recent human evidence. Fifty-three participants with NTG received nicotinamide at 1 g/day for six weeks followed by 2 g/day for six weeks, or placebo, with crossover. Nicotinamide produced significantly greater improvements in the photopic negative response and B-wave amplitude, but there were no significant between-group differences in mean deviation, pattern standard deviation or visual-field index after 12 weeks. The study provides evidence of an effect on retinal electrophysiological function, but not evidence that nicotinamide slows visual-field progression [44].

The field has consequently moved toward longer trials designed specifically to test progression. A phase III randomized, placebo-controlled study of nicotinamide plus pyruvate in treated open-angle glaucoma is designed over 21 months, with a primary endpoint incorporating change in the central 10° visual field and/or spectral-domain OCT structural change. The study also includes metabolomic, transcriptomic and proteomic assessments and seeks to identify factors associated with response to metabolic neuroprotection [58].

Safety remains an important consideration. The American Glaucoma Society and American Academy of Ophthalmology issued a joint position statement in 2025 regarding nicotinamide use for glaucoma neuroprotection, reflecting concerns about the high doses used in some clinical studies and the potential for systemic toxicity. Routine use of high-dose nicotinamide for glaucoma neuroprotection is not currently supported outside appropriately monitored clinical research [45].

#### 3.1.5. Citicoline and Mitochondrial Support

Citicoline has been investigated as a potential neuroprotective and neuroenhancing treatment, with proposed effects on membrane phospholipid metabolism and neuronal function. Small clinical studies have reported changes in electrophysiological measures, but evidence that citicoline slows glaucoma progression remains inconclusive. A 2023 systematic review identified 10 clinical studies involving 424 patients and found no significant overall benefit of citicoline compared with control for IOP, visual-field mean deviation, RNFL thickness or PERG amplitude. The authors concluded that there was insufficient evidence to support citicoline as a treatment that slows glaucoma progression [59].

Mitochondrial support with coenzyme Q10 and vitamin E has similarly generated physiological and electrophysiological signals. In a clinical study of patients with open-angle glaucoma, treatment was associated with improvements in pattern electroretinography and visual-evoked potential parameters, but these findings do not establish a reduction in structural or functional progression [48]. More recent clinical work has continued to investigate this approach, but the evidence remains insufficient to establish CoQ10/vitamin E as a proven neuroprotective treatment [60].

#### 3.1.6. Neurotrophic-Factor Delivery

Neurotrophic-factor delivery represents a different strategy, aiming to support RGC survival rather than modify IOP or a single downstream injury pathway. Ciliary neurotrophic factor (CNTF) has substantial experimental evidence for promoting RGC survival, but sustained delivery to the retina and optic nerve is a major translational challenge. NT-501 is an encapsulated-cell implant designed to provide sustained intravitreal delivery of CNTF.

Direct intravitreal delivery of recombinant BDNF is another experimental neurotrophic approach that predates current gene- and cell-based strategies. In rat models, intravitreal BDNF has shown partial RGC protection after experimentally elevated IOP, while earlier optic-nerve injury work demonstrated protection of axonal die-back but not prevention of rapidly progressing RGC degeneration. These findings support the biological rationale for BDNF delivery but also illustrate the limitations of protein replacement: effects depend on injury model, timing and repeated administration, and the cited studies are preclinical rather than evidence of an established human glaucoma treatment [61,62].

Clinical development has progressed to randomized phase II studies. The ClinicalTrials.gov record for NCT02862938 describes a randomized, sham-controlled, masked phase II study of NT-501 in glaucoma with a planned enrollment of 54 participants and a primary completion date of August 2025; however, no results were posted in the registry record reviewed for this manuscript. Because recruitment-status information has not been consistent across publicly available institutional and registry sources, current recruitment status is not used here as evidence of programme progress. A second randomized, sham-controlled phase II study, NCT04577300, evaluates single versus dual NT-501 implantation, with visual-field mean deviation at 12 months as the primary outcome. No efficacy results are currently available in the public record [49,50].

The absence of posted efficacy results means that NT-501 should remain classified as investigational. The approach is biologically distinct from metabolic rescue or excitotoxicity modulation, but the clinical question remains the same: whether sustained neurotrophic support can translate into measurable preservation of visual function in glaucoma.

#### 3.1.7. Where Are We Now?

Clinical development of glaucoma neuroprotection has moved well beyond a purely experimental concept. Human studies have tested approaches targeting excitotoxicity, neurotrophic support, vascular regulation, mitochondrial function and cellular metabolism, while newer programmes are incorporating more sensitive functional and structural endpoints. No pressure-independent treatment, however, has yet established a clinically validated effect on glaucomatous progression sufficient to support routine use as neuroprotection. The 2026 literature describes a field moving toward metabolic therapies, sustained neurotrophic-factor delivery, functional enhancement and, increasingly, biomarker- and precision-medicine approaches.

The challenge is not simply to identify another biologically plausible target. A successful neuroprotection programme must show that modifying the target produces a reproducible change in clinically meaningful structural or functional outcomes. This is particularly difficult in glaucoma because progression can be slow, standard automated perimetry and OCT have measurement variability, and patients differ substantially in their susceptibility to damage and the rate at which they progress. Contemporary trial methodology is consequently placing greater emphasis on identifying faster progressors, improving endpoint sensitivity and combining structural and functional measures to increase the ability to detect a treatment effect [41,63,64].

## 4. The Endpoint Problem

The difficulty becomes most apparent when neuroprotective strategies are tested clinically. The outcome of greatest relevance to patients is preservation of visual function, but glaucomatous progression is generally slow and conventional perimetric measurements are subject to substantial test–retest variability. Neuroprotection trials must therefore detect a treatment-related difference in progression within a feasible follow-up period while maintaining a clear relationship between the endpoint and preservation of vision.

Visual-field testing remains particularly important because it directly measures functional loss and is an established outcome measure in glaucoma clinical trials. Conventional event-based analyses classify an eye as progressed when a predefined pattern of deterioration is reached. Trend-based approaches instead estimate the rate of change over time and can therefore use information from repeated measurements before an eye reaches a progression threshold. In a large longitudinal analysis of 3352 patients with at least 10 visual-field examinations and a median follow-up of 11 years, Montesano et al. compared trend-based methods with FDA-compatible event-based criteria. In simulated 2-year trials, linear mixed-model and time-to-progression approaches based on mean deviation had greater statistical power than the event-based methods for detecting simulated treatment effects that reduced the risk of long-term progression [65]. Importantly, these were simulations based on observed long-term progression data and do not establish trend-based analysis as a regulatory endpoint.

Independent work has provided further support for using the rate of visual-field change as a potential endpoint. De Moraes et al. evaluated whether mean-deviation slopes calculated over approximately two years were predictive of subsequent FDA-compatible visual-field progression. Eyes with rapid MD decline over the two-year period were approximately ten times more likely to subsequently reach one of the progression criteria evaluated. The findings support the potential use of short-term MD slope as an indicator of future progression, but the study was retrospective and does not establish that MD slope itself is an accepted surrogate for long-term visual preservation [66].

More recent work has moved beyond the use of global MD alone. In 2026, da Costa et al. developed and externally validated an artificial-intelligence model designed to identify the visual-field locations most likely to deteriorate using baseline standard automated perimetry. The study included 82,449 visual-field tests from 14,237 eyes across three independent datasets from Bascom Palmer, Duke and the University of Washington. The model identified five high-risk locations for each eye (“High-5”). In progressing eyes, the High-5 locations deteriorated at approximately twice the rate of global MD, with mean slopes of −2.05 to −2.32 dB/year compared with −0.93 to −1.14 dB/year for MD across the validation cohorts. The High-5 measure also showed strong discrimination between progressing and non-progressing eyes. The authors proposed this as a proof-of-concept framework for more sensitive functional endpoints in neuroprotection trials, rather than as an established regulatory endpoint [67].

The significance of this approach is that it shifts the endpoint question from how quickly global visual-field indices change to whether the most informative regions of the visual field can be identified prospectively. High-5 remains investigational, but it illustrates how patient-level baseline information could potentially be used to enrich a trial for locations with a greater likelihood of measurable progression. This may increase sensitivity to treatment effects without requiring the entire visual field to deteriorate at the same rate.

This directly addresses the prospective-evaluation question: the published High-5 study was retrospective and externally validated, not a prospective randomized treatment trial.

The High-5 study was based on retrospective longitudinal visual-field datasets and external validation of the prediction model; it did not prospectively randomize patients to a treatment and test whether a treatment-induced High-5 change predicted preservation of vision. Its demonstrated ability to identify locations with faster natural progression is therefore evidence for prognostic discrimination and endpoint development, not evidence that High-5 increases the power of a prospective neuroprotection trial. A prospective treatment study would need to prespecify the High-5 endpoint, its analysis, handling of missing or unreliable fields, and the treatment effect of interest before its statistical efficiency and clinical interpretability could be established [67].

OCT provides a complementary approach by measuring structural changes in the retinal nerve fibre layer and macular ganglion-cell tissue. Because structural loss may precede or accompany detectable functional deterioration, OCT has been investigated as an additional endpoint for glaucoma trials. A simulation study using longitudinal data from 192 eyes of 121 patients found that combining visual-field and OCT measures reduced estimated sample-size requirements by approximately 31–33% compared with visual-field endpoints alone for the treatment effects examined [68]. The authors emphasized that the estimates were dependent on the characteristics of the population, treatment effect and trial design and should not be interpreted as a universal reduction in sample size.

The more recent evidence, however, argues against assuming that adding OCT will always improve trial efficiency. A 2026 simulation study using longitudinal structure–function data found that the rate of MD change had greater statistical power than the rate of circumpapillary RNFL change for detecting a simulated 30% neuroprotective effect in early-to-moderate glaucoma. Combining the two measures produced only a marginal improvement over MD alone in that analysis [69]. These findings reinforce an important point: the value of an endpoint depends on its rate of change, variability, relationship to meaningful progression and the population and trial design in which it is used; OCT should not automatically be assumed to be more sensitive than visual field testing.

This distinction matters in neuroprotection trials. IOP remains an important pharmacodynamic measure and should be controlled or accounted for, but a reduction in IOP cannot by itself demonstrate an IOP-independent neuroprotective effect. A treatment intended to protect RGCs independently of further pressure reduction must ultimately demonstrate an effect on neuronal structure or function. Current approaches therefore favour functional endpoints, structural endpoints, or combinations of the two, while recognising that a surrogate requires evidence that a treatment-induced change reliably predicts a corresponding effect on a patient-important clinical outcome [70].

The regulatory distinction is more nuanced than a simple separation between ‘functional endpoints for approval’ and ‘surrogates for early trials’. Regulatory agencies distinguish clinical outcome assessments from biomarkers and evaluate surrogate endpoints according to their evidentiary context. A biomarker can be used as a candidate endpoint during development without being a validated surrogate, while a validated or otherwise accepted surrogate may support efficacy conclusions in an appropriate context. For glaucoma neuroprotection, the central problem is that no structural or cellular biomarker discussed here has yet demonstrated the treatment-effect validation required to substitute reliably for clinically meaningful visual preservation. This is why structural and biological measures may be highly informative in dose-finding and proof-of-mechanism studies while functional preservation remains critical for demonstrating clinical benefit [31,71].

The problem is further illustrated by biomarkers of RGC injury. Detection of Apoptosing Retinal Cells (DARC) enables in-vivo imaging of apoptotic retinal cells using fluorescently labelled annexin. First-in-human work demonstrated the feasibility of detecting individual apoptotic retinal cells in patients with glaucoma [72]. Subsequent work using automated, convolutional-neural-network-assisted DARC analysis found higher baseline DARC counts in glaucoma eyes that subsequently demonstrated OCT-defined RNFL progression, with an AUC of 0.89 in the study population [73]. These findings support DARC as a promising biomarker of retinal cell apoptosis and potential risk of progression, but an association with subsequent progression does not establish DARC as a validated surrogate endpoint for preservation of visual function.

The distinction between a biomarker, a clinical endpoint and a surrogate endpoint is fundamental. A biomarker may reflect a biological process associated with disease activity; a clinical endpoint measures an outcome that is directly meaningful to the patient; and a surrogate endpoint requires evidence that an intervention-induced change in the surrogate reliably predicts the corresponding effect on the clinical outcome. In glaucoma, OCT measurements, visual-field indices, electrophysiological measures and emerging cellular biomarkers can provide complementary information about disease activity and treatment response, but none should automatically be regarded as a validated surrogate for long-term visual preservation [70].

These sample-size and power estimates should therefore be interpreted as statistical simulations rather than evidence that a particular endpoint will improve the ability of a prospective treatment trial to demonstrate neuroprotection. Simulation can estimate the consequences of variability, progression rate, visit frequency and an assumed treatment effect under specified data-generating assumptions; it cannot demonstrate that the assumed treatment effect exists, that the endpoint is causally related to treatment benefit, or that a statistically efficient endpoint will translate into clinically meaningful preservation of vision. Prospective randomized trials remain necessary to establish treatment effects, while external validation and, for a surrogate endpoint, evidence linking treatment-induced changes in the surrogate to patient-important outcomes are required for stronger claims of surrogate validity [31,70,71].

The proposed evidentiary roles of these measures are summarized in Table 5.

For clarity, the measures discussed here should not be assigned the same evidentiary role. Mean deviation (MD) slope is a longitudinal functional outcome measure and candidate clinical trial endpoint because it quantifies change in visual function; it can also be used prognostically to estimate future progression. It is therefore different from OCT or DARC, which are indirect structural or biological measures. An MD slope treatment effect would constitute direct evidence of a functional treatment effect, but the magnitude, durability and clinical relevance of that effect still need to be established. OCT measures such as RNFL or macular ganglion-cell thickness are structural biomarkers and can also be used as clinical trial endpoints; their ability to serve as surrogate endpoints for visual preservation remains unvalidated. High-5 is best regarded at present as an investigational, patient-specific functional endpoint and potential enrichment strategy derived from visual-field data. DARC is a biological biomarker of retinal cell apoptosis with prognostic potential because baseline DARC measures have been associated with subsequent structural progression, but it has not been shown to predict a treatment-induced preservation of visual function. Accordingly, the present evidence supports a hierarchy of functional endpoints, candidate structural/biological biomarkers and investigational enrichment approaches, rather than treating these measures as interchangeable surrogate endpoints [67,70,72,73].

The endpoint challenge is not simply to identify a measure that changes earlier than visual field. The measure must be sufficiently sensitive to detect a treatment effect, sufficiently reproducible to distinguish that effect from measurement variability, and sufficiently linked to clinically meaningful progression for the result to be interpretable. Recent work on trend-based analyses, structure–function models and AI-guided selection of vulnerable visual-field locations reflects this shift. The field is moving from asking whether a single measurement can serve as a surrogate for vision toward identifying combinations of measurements and analytical methods that can detect meaningful preservation of RGC structure and function within a feasible clinical-trial duration.

The evidentiary standard also differs according to what is being claimed. For MD slope, evidence of a genuine neuroprotective effect would require a prespecified treatment-related difference in longitudinal functional decline that is reproducible, clinically interpretable, and sustained over an adequate period, with appropriate control of IOP and other sources of bias. For OCT, High-5 or DARC, a surrogate claim requires additional evidence that an intervention-induced change in the candidate measure predicts the corresponding treatment-induced effect on clinically meaningful visual function. At minimum, this requires adequate analytical reliability, prespecified analyses, reproducible longitudinal behaviour, biological plausibility, and evidence from randomized treatment data linking the change in the candidate measure to the treatment effect on visual function. Association with natural-history progression alone is insufficient for treatment-surrogate validation [31,71].

Endpoint development is also inseparable from trial population and design. Selecting patients with measurable progression and sufficiently reliable longitudinal measurements can materially increase statistical power. In a simulation study of 2804 patients, selecting participants with lower inter-test visual-field variability reduced estimated sample size by 38% and 49% for simulated 30% and 50% treatment effects, respectively, compared with random selection [74]. The ability to demonstrate neuroprotection therefore depends not only on the biological activity of the intervention, but also on who is enrolled, what is measured, how often it is measured, and how progression is analysed.

This also argues against assuming that a novel biomarker is necessarily the most practical route to greater trial efficiency. Patient selection, measurement frequency, quality control and longitudinal statistical modelling can each change the signal-to-noise ratio of a conventional endpoint. In simulation, selecting eyes with lower inter-test VF variability reduced estimated sample size substantially, while other work has shown that test pattern and frequency influence the time required to detect progression. These findings support a development strategy in which enrichment and improved measurement are considered alongside, rather than automatically replaced by, new biomarkers [74].

## 5. Current Investigational Pipeline

The clinical pipeline for treatment beyond IOP lowering has become more differentiated. Metabolic approaches have progressed furthest in terms of randomized clinical development, while neurotrophic-factor delivery has reached phase II investigation. Other approaches, including functional enhancement and vascular or metabolic modulation, remain at earlier stages of clinical evaluation. Gene- and cell-based strategies are also advancing, but their level of maturity differs substantially: NT-501 provides an example of a cell-based neurotrophic approach already being tested in patients, whereas gene-based approaches to RGC neuroprotection remain predominantly preclinical.

### 5.1. Metabolic Approaches

Nicotinamide is currently one of the most advanced pharmacological approaches to pressure-independent neuroprotection. The rationale is based on the role of NAD metabolism and mitochondrial function in maintaining RGC bioenergetic reserve. Two phase III programmes are now evaluating whether metabolic supplementation can slow functional or structural progression in patients receiving standard IOP-lowering treatment.

The UK NAMinG trial is a multicentre, randomized, double-masked, placebo-controlled phase III study evaluating nicotinamide 3 g/day in adults with early-to-moderate open-angle glaucoma. Recruitment closed in April 2026 after 378 participants had been randomized, and follow-up is continuing through 2028. The primary clinical question is whether nicotinamide slows visual-field loss over 27 months; the study also incorporates mechanistic assessments of mitochondrial function and NAD-related biomarkers (NAMinG, NCT05405868; ISRCTN16435278).

A separate phase III trial, NIC-OAG, is evaluating nicotinamide 3 g/day combined with calcium pyruvate 1 g/day in treated open-angle glaucoma. The 21-month, double-masked randomized trial uses a composite primary endpoint incorporating central 10° automated perimetry and/or spectral-domain OCT, together with metabolomic, transcriptomic and proteomic assessments. Importantly, the study also prospectively evaluates factors associated with responsiveness to metabolic neuroprotection, directly addressing the possibility that treatment effects may differ between patients [58].

The clinical evidence remains preliminary. A 2026 systematic review of randomized trials concluded that the current evidence does not support nicotinamide for improving mean deviation in routine clinical practice, citing small sample sizes, short follow-up and methodological limitations. The ongoing phase III studies are therefore important because they move the field from short-term electrophysiological or exploratory signals to adequately powered longitudinal assessments of structural and functional progression [75].

### 5.2. Topical Neurofunctional Approaches

BL1107 illustrates both the potential and the difficulty of translating a neurofunctional hypothesis into a clinical treatment. BL1107 is a topical alpha-2 adrenergic agonist developed by Bausch + Lomb with the aim of improving visual function through proposed neuroprotective and neuroenhancement effects. The phase II study enrolled 159 adults with POAG or ocular hypertension and used change from baseline in visual-field mean deviation at Day 28/exit as the primary endpoint; low-luminance BCVA and IOP were secondary measures. The study did not meet its primary endpoint. In July 2026, Bausch + Lomb announced that it would discontinue development of BL1107 as a topical glaucoma treatment [52,76].

BL1107 is relevant to the endpoint problem described above. The study was designed to test a measurable improvement in visual function over a short treatment period, but the phase II result did not reproduce the earlier signal. BL1107 should therefore no longer be described as an ongoing glaucoma pipeline programme; it is better regarded as a recent example of a neurofunctional strategy that did not progress following a negative phase II result.

### 5.3. Neurotrophin-Based Therapies

NT-501 represents a different approach, using an encapsulated-cell implant to provide sustained intravitreal delivery of ciliary neurotrophic factor (CNTF). The strategy is intended to provide prolonged trophic support to the retina without requiring repeated intravitreal administration.

An earlier phase I study in POAG demonstrated that NT-501 was tolerated over 18 months and reported structural and functional changes in treated eyes, including changes in visual-field indices and RNFL thickness. However, the study was small and descriptive and did not provide controlled evidence of efficacy [51].

The programme subsequently progressed to randomized phase II investigation. NCT02862938 is a randomized, sham-controlled phase II study of NT-501 in glaucoma with a planned enrollment of 54 participants and a primary completion date of August 2025. No results were posted in the registry record reviewed for this manuscript. NCT04577300 is a second randomized, sham-controlled phase II study evaluating single versus dual NT-501 implantation, with visual-field MD at 12 months as the primary endpoint and VFI and pointwise linear regression as secondary functional measures. No efficacy results are currently available in the public record.

NT-501 has progressed further clinically than most neuroprotective approaches, but clinical efficacy remains unestablished. The approach remains investigational.

## 6. Gene- and Cell-Based Strategies

Gene and cell therapies offer the possibility of sustained biological activity and address a different limitation from conventional pharmacological treatment. For glaucoma, however, the therapeutic objective is particularly demanding. An intervention intended to protect RGCs must reach the relevant neuronal population, produce an appropriate and sustained biological effect, avoid unacceptable local or systemic toxicity, and preserve neuronal function over the long period during which glaucomatous degeneration occurs.

Gene therapy has been investigated experimentally through several mechanisms, including enhancement of neurotrophic signalling, modulation of apoptosis and mitochondrial function, and regulation of neuroinflammatory pathways. AAV2-based delivery of BDNF has been shown to improve RGC survival in experimental models, but sustained BDNF signalling can be limited by downregulation of its TrkB receptor. AAV constructs designed to enhance both TrkB and mature BDNF signalling have therefore been developed as an alternative strategy [77].

The experimental gene-therapy literature is also expanding beyond the canonical BDNF/TrkB pathway. Preclinical gene-therapy studies have investigated several survival and stress pathways beyond BDNF/TrkB. AAV-mediated activation of the ERK1/2 pathway protected RGCs in experimental ocular hypertension; more recent AAV-mediated RXRα overexpression preserved RGC structure and function through PI3K/Akt/GSK3β signalling; and a 2026 CaMKII-based gene-therapy study reported protection of RGCs and visual function in mouse models of ischemic injury and congenital glaucoma. These findings illustrate the breadth of experimental pathway targeting, but they remain preclinical and should not be presented as evidence of clinical efficacy [78,79,80]. The most advanced human gene-therapy examples in glaucoma should also be distinguished by mechanism: GVB-2001 is an intracameral AAV2-based RhoA-targeting programme intended to lower IOP in POAG, not a clinical test of pressure-independent RGC neuroprotection [81].

More recent preclinical work has continued this approach. A 2025 study evaluated a bicistronic AAV vector expressing human TrkB and mature BDNF in experimental models of optic nerve injury and ocular hypertension. The treatment produced sustained transgene expression and preserved RGC survival and visual function in the experimental models. These findings strengthen the preclinical rationale for combined TrkB/BDNF signalling, but they remain experimental evidence and do not establish clinical efficacy in glaucoma [82].

Gene therapy for pressure-independent RGC neuroprotection has not yet reached established human clinical development. A 2026 clinical study of GVB-2001, for example, represents an investigational gene therapy delivered intracamerally for POAG, but its target is the RhoA pathway in the anterior segment and its objective is IOP reduction rather than RGC neuroprotection. It should therefore not be presented as evidence of clinical gene therapy for neuroprotection (NCT06921317) [81].

Cell-based approaches face a different set of challenges. Experimental studies of mesenchymal stromal cells have reported preservation of RGCs and retinal structures, with proposed effects involving trophic and immunomodulatory signalling. A 2025 systematic review and meta-analysis of experimental glaucoma models found evidence of increased RGC counts, nerve-fibre-layer thickness and retinal neurotrophic-factor expression following stem-cell transplantation [83]. However, the evidence was entirely preclinical, and the authors identified unresolved issues concerning cell source, delivery, targeting and long-term safety.

The distinction between cell-based delivery of a neuroprotective factor and replacement of lost RGCs is important. NT-501 is being tested clinically because the encapsulated cells are used as a sustained source of CNTF rather than as replacement neurons. True neuronal replacement would require transplanted cells to survive, differentiate appropriately, integrate into the retinal circuitry, extend axons through the optic nerve and establish appropriate connections with central visual targets. That remains a substantially more difficult regenerative objective.

The same translational requirement applies to both approaches as to pharmacological neuroprotection: biological activity is not sufficient. The intervention must ultimately demonstrate preservation—or, for regenerative approaches, meaningful restoration—of clinically relevant visual function.

## 7. Systemic Comorbidity, Biological Heterogeneity and Trial Design

The variability in glaucoma progression is not explained by IOP alone. Systemic and ocular factors may modify susceptibility to glaucomatous injury, particularly vascular and metabolic factors. Hypertension, hypotension, diabetes, dyslipidaemia, obstructive sleep apnoea, chronic kidney disease and other systemic conditions have all been investigated in relation to glaucoma, although the strength and consistency of the evidence differ between conditions [84].

Vascular factors are particularly relevant to trial interpretation. A 2025 prospective cohort analysis from the Vascular Imaging in Glaucoma Study found relationships between systemic blood-pressure measures and rates of visual-field change, supporting the importance of systemic haemodynamic factors in understanding individual progression. These findings do not establish that modifying systemic blood pressure will prevent glaucoma progression, but they reinforce the biological heterogeneity that exists within apparently similar glaucoma populations [85].

This heterogeneity creates a particular challenge for neuroprotection trials. A treatment directed at mitochondrial dysfunction, vascular dysregulation or neurotrophic support may not be expected to have the same effect in every patient. Conversely, a modest treatment effect in a biologically responsive subgroup may be diluted when the intervention is evaluated in an unselected population.

Recent work is moving toward better characterization of individual progression trajectories and treatment responsiveness. Structural measurements, visual-field history and clinical variables can be combined to predict future progression, and deep-learning models have demonstrated the ability to use longitudinal OCT information to predict subsequent visual-field deterioration [86].

At present, no molecular biomarker has been prospectively validated as defining a glaucoma subgroup with a reproducibly different rate of progression and a differential response to a neuroprotective treatment. Genetic, metabolic, vascular and multi-omic characteristics are being investigated, and current trials such as NIC-OAG incorporate prospective metabolomic, transcriptomic and proteomic assessments, but these measurements are exploratory until they are prospectively replicated and shown to modify treatment effect. A prognostic association alone is not sufficient to establish a predictive biomarker of treatment response.

The implications for neuroprotection extend beyond simply increasing sample size. Baseline progression rate, structural reserve, vascular and metabolic characteristics, concomitant IOP-lowering treatment and the reliability of longitudinal functional measurements may all influence the ability to detect a treatment effect. The emerging use of multi-omics, biomarkers and AI-based prediction models reflects increasing interest in identifying biologically and clinically meaningful subgroups rather than treating glaucoma as a homogeneous disease entity [41,87].

This approach also changes how clinical trials should be designed. If a treatment is expected to slow progression rather than produce an immediate change in vision, participants need sufficient baseline damage and a sufficiently measurable rate of progression for a treatment effect to emerge within the study period. The recent development of the NAMinG and NIC-OAG trials illustrates this shift: both maintain standard IOP-lowering treatment while prospectively evaluating whether a metabolic intervention changes longitudinal visual or structural outcomes, and NIC-OAG additionally incorporates biological measurements intended to identify factors associated with treatment response.

An optimal baseline MD range cannot be defined universally for neuroprotection trials. The selected population should have sufficient residual visual function and structural reserve to allow meaningful detection of further deterioration, while also having a sufficient rate of progression for a treatment effect to become detectable within the planned follow-up period. This balance will depend on the endpoint used. In advanced glaucoma, OCT measures may be affected by structural floor effects, while visual-field variability and the ability to detect further functional progression also vary with disease severity and testing strategy. Baseline disease severity should therefore be considered together with the expected rate of progression, residual functional and structural reserve, measurement reliability, and the characteristics of the selected endpoint, rather than using baseline MD alone as a universal enrichment criterion [74].

## 8. Conclusions

IOP lowering remains the only treatment strategy with established evidence for reducing the risk of glaucomatous progression. This applies to patients with both elevated and normal-range IOP: the clinical evidence in NTG demonstrates that further IOP reduction can reduce progression risk even when untreated IOP is not consistently elevated. The persistence of progression in some patients despite IOP lowering nevertheless provides a rationale for investigating mechanisms that influence RGC susceptibility, including mitochondrial function, neurotrophic support, vascular regulation and cellular stress.

The clinical development history of neuroprotection shows how difficult it is to translate these mechanisms into meaningful preservation of human vision. Memantine failed to demonstrate benefit in two large phase III trials, the apparent visual-field signal with brimonidine was complicated by differential treatment discontinuation, vascular interventions have not established prevention of progression, and the recent phase II failure of BL1107 shows that an early signal of visual-function improvement may not be reproduced in a larger study.

The field has nevertheless changed substantially. Nicotinamide has progressed into multiple phase III programmes, including the UK NAMinG trial and the NIC-OAG trial, while NT-501 has progressed through randomized phase II development as a sustained neurotrophic-factor delivery system. Gene-based approaches remain predominantly preclinical for pressure-independent neuroprotection, but newer AAV strategies are becoming increasingly sophisticated in their targeting of RGC survival pathways.

The definition of a successful neuroprotection trial is also becoming more sophisticated. Recent work has moved from reliance on conventional global visual-field measures toward trend-based analyses, combined structural and functional endpoints, identification of high-risk visual-field locations using AI, and biological characterization of treatment response. The aim is not simply to demonstrate a change in a biomarker but to establish that the change predicts slower structural or functional deterioration that is meaningful to patients.

The remaining challenge is both biological and methodological. A potentially effective treatment may produce only a modest effect against a slowly progressing disease, and that effect may be diluted by heterogeneity in progression rate, disease stage, systemic factors and underlying biological susceptibility. Future neuroprotection trials will need to address these factors prospectively through appropriate patient selection, sensitive and clinically meaningful endpoints, longitudinal analysis and, where justified, biological enrichment.

Until an intervention targeting a pressure-independent mechanism demonstrates reproducible preservation of clinically meaningful visual function in adequately controlled human trials, these approaches should remain distinguished from established IOP-lowering treatment. The next stage of glaucoma drug development is not simply to identify another neuroprotective target but to demonstrate that modifying that target changes the trajectory of disease in the patients most likely to benefit.

## Figures and Tables

**Table 2:** Sustained-release delivery platforms: potential advantages and key translational issues.

Platform	Potential Advantage	Key Translational Issue
Intracameral implant	Long-duration, local exposure; reduced drop burden	Endothelial safety, repeat dosing, device-related risks and reversibility
Subconjunctival depot/injectable system	Prolonged exposure without daily instillation	Release kinetics, local inflammation, dose control and reversibility
Punctal/insert/ring systems	Reduced administration frequency	Retention, comfort, drainage and reproducibility of delivered dose
Drug-eluting contact lens	Continuous topical delivery with potential adherence benefit	Comfort, optical performance, release control and patient acceptance
Microsphere/nanoparticle/hydrogel systems	Potentially tunable release and tissue targeting	Manufacturing, sterility, particle tolerability and predictable in-vivo release

**Table 3:** Sustained-release IOP-lowering products.

Platform	Drug	Delivery	Status
Durysta	Bimatoprost	Biodegradable intracameral implant	FDA-approved; single implant per eye [20,26]
iDose TR	Travoprost	Non-biodegradable intracameral implant anchored at the angle	FDA-approved; re-administration permitted in patients meeting specified endothelial cell-density criteria [27,29]
OTX-TIC/paxtrava	Travoprost	Intracameral hydrogel	Phase 2 completed; next development steps under evaluation [30]
ENV515	Travoprost	Intracameral sustained-release implant	Phase 2 completed; no current development activity identified [31]
Other platforms	Various	Drug-eluting IOLs, punctal systems, rings, contact lenses	Investigational/mixed development

**Table 5:** Candidate roles and current evidentiary status.

Measure	Current Role	What Is Supported	What Is Not Yet Established
MD slope	Functional clinical outcome measure/candidate trial endpoint; prognostic measure	Longitudinal progression rate and association with future progression	Validation as a treatment surrogate is not established; the clinically meaningful interpretation of a short-term slope difference also depends on trial duration and magnitude
OCT RNFL/ganglion-cell measures	Structural biomarker and candidate trial endpoint	Structural change can accompany or precede functional loss; useful for longitudinal assessment	That treatment-induced structural change reliably predicts patient-important visual benefit
High-5	Investigational functional endpoint/enrichment approach	Baseline data can identify locations with faster subsequent deterioration	Prospective treatment-effect benefit or regulatory surrogate validity
DARC	Candidate biological/prognostic biomarker	Apoptotic-cell signal and association with subsequent structural progression	Prediction of treatment-induced preservation of visual function

## Data Availability

The original contributions presented in this study are included in the article. Further inquiries can be directed to the corresponding author.

## Abbreviations

AAV, adeno-associated virus; BDNF, brain-derived neurotrophic factor; cGMP, cyclic guanosine monophosphate; CNTF, ciliary neurotrophic factor; EP2/EP3, prostaglandin E2 receptor subtypes 2/3; FP, prostaglandin F receptor; IOP, intraocular pressure; MD, mean deviation; NAD+, nicotinamide adenine dinucleotide; NMDA, N-methyl-D-aspartate; NO, nitric oxide; NTG, normal-tension glaucoma; OAG, open-angle glaucoma; OCT, optical coherence tomography; PERG, pattern electroretinogram; PhNR, photopic negative response; POAG, primary open-angle glaucoma; RGC, retinal ganglion cell; ROCK, Rho-associated kinase; VF, visual field; VFI, visual field index; VEP, visual evoked potential.
